# Instrumentation on Multi-Scaled Scattering of Bio-Macromolecular Solutions

**DOI:** 10.3390/ijms160510016

**Published:** 2015-05-04

**Authors:** Benjamin Chu, Dufei Fang, Yimin Mao

**Affiliations:** Chemistry Department, Stony Brook University, Stony Brook, New York, NY 11794-3400, USA; E-Mails: Dufei.fang@stonybrook.edu (D.F.); yimin.mao@mse.gatech.edu (Y.M.)

**Keywords:** laser/X-ray scattering, photon cross correlation spectroscopy, size, structure, dynamics

## Abstract

The design, construction and initial tests on a combined laser light scattering and synchrotron X-ray scattering instrument can cover studies of length scales from atomic sizes in Angstroms to microns and dynamics from microseconds to seconds are presented. In addition to static light scattering (SLS), dynamic light scattering (DLS), small angle X-ray scattering (SAXS) and wide angle X-ray diffraction (WAXD), the light scattering instrument is being developed to carry out studies in mildly turbid solutions, in the presence of multiple scattering. Three-dimensional photon cross correlation function (3D-PCCF) measurements have been introduced to couple with synchrotron X-ray scattering to study the structure, size and dynamics of macromolecules in solution.

## 1. Introduction

### 1.1. Motivation

Investigations on the changes in sizes, size distributions, and structures of bio-macromolecular aggregates in solution, such as the formation of Aβ peptides and complexes based on their interactions with inhibitors (or *C*-terminal fragments (CTFs) of Aβ42), are challenging issues. While many physical techniques have been used to elucidate (bio-) macromolecular aggregates and their corresponding complexes, more probes that can provide fundamental information in the structure and dynamics of such systems should be useful. In this article, we describe the design, construction, and initial tests of an instrument using a combination of laser and synchrotron X-rays to investigate the structure and dynamics of macromolecular solutions or colloidal suspensions over a broad range of time and length scales. The description also includes a brief summary of the rationale on choosing the specific designs for the instrumentation, as there are different pathways available.

Laser light scattering (LLS) is a powerful tool to investigate sizes, size distributions, and dynamic properties of macromolecules in solution or colloidal particles in suspension [1,2]. However, the practical application of conventional LLS is limited to *dilute* solutions, in the absence of multiple scattering. While conventional schemes, including reduction of light path, usage of very thin light scattering sample cells, and/or closer matching of the refractive index of solvent with that of the macromolecular solute, have been used [3], there are many instances that seek single scattering information in the presence of multiple scattering where conventional schemes are insufficient. For example, in order to understand the reaction mechanisms associated with aggregation processes, it is desirable to consider other alternatives. It should be noted that, in principle, multiple scattering can be modeled theoretically, but due to its nature, being related to the many-body problem, the treatment of higher-order scattering (e.g., beyond triple scattering) becomes formidably complicated [4]. Nevertheless, we do want to take advantage of the feasibility to reduce the light path in a practical way in the current instrumentation setup.

### 1.2. Classical Approaches to Reduce Multiple Scattering

The possibilities of reducing the cell diameter for a cylindrical cell or the cell thickness for a flat cell are limited; usually the lower limit is in the order of several millimeters for a cylindrical cell or a few hundred microns for a flat sample cell [1,3]. The refractive index matching approach involves matching the refractive index of the solvent (or suspending medium) with that of the solute (or the suspended particles), yielding to a reduction in the overall scattering power. This method has practical difficulties in preparing solutions because it often becomes a challenging undertaking to satisfy both the matching of refractive index and the chemical requirements between the solvent and the solute [5,6,7]. There is also a fiber-optic approach that has worked successfully, with commercial probes being available, but it is mainly limited to the back scattering geometry [8,9,10].

The treatment of multiple scattering could also be approached from the very high concentration end, assuming that light is being randomly scattered so many times in the scattering media that its trajectory can be approximated by a random walk [11,12]. The diffusing-wave spectroscopy (DWS) requires a very high concentration of the measured sample and has not been able to generate certain types of particle information, such as polydispersity [13].

### 1.3. Cross Correlation Function Approach

We now present an exciting method that can simultaneously use three-dimensional photon cross correlation function (3D-PCCF) [3,14,15,16,17,18,19,20] measurements in combination with synchrotron X-ray scattering. 3D-PCCF can experimentally extract single scattering behavior from a reactive bio-macromolecular solution in the presence of multiple scattering. The dynamical and structural results, including size and size distribution, together with shape and/or internal structures, as a function of reaction time, under different experimental conditions, such as pH, ionic strength, and temperature, can be analyzed in combination with synchrotron small angle X-ray scattering and wide angle X-ray diffraction (SAXS/WAXD), to further elucidate the nature of aggregates (or complex formation) during different stages of the aggregation (or complex formation) process.

In 3D-PCCF, two laser beams define the scattering volume, which can be quite small (with beam diameters, for example, at around 100 microns). Instead of the temporal auto-correlation function generated in a single-beam dynamic light scattering (DLS) experiment, the time-dependent scattered intensities are captured by two individual detectors and a temporal cross-correlation function is computed in 3D-PCCF. The scattering geometry of the 3D-PCCF experiment has to be carefully designed such that the two scattering experiments taking place simultaneously using the two laser-detector groups have exactly the same scattering vector *q*, with the magnitude of *q* (=4π*n*λ^−1^sin(θ/2) with θ, *n* and λ being, respectively, the scattering angle, the refractive index in the scattering medium, and the laser incident wavelength). Only then, the cross-correlation function of their single scattering events is, apart from an efficiency factor, equivalent to the autocorrelation function of a single-beam DLS experiment and can be evaluated in the same fashion to generate particle size information. Multiple scattering events are un-correlated and can be subtracted as background. Thus, this approach allows us to separate multiple scattering effects *experimentally* from the desired single scattering information. The approach is effective for *mildly* turbid solutions since the amount of multiple scattering will increase and, thus, the efficiency of the approach will decrease with increasing turbidity of the solution. Furthermore, the measurement is not limited to only a small range of scattering angles when choosing a cylindrical sample cell. Therefore, the set-up can also be used for general laser light scattering measurements [19,21].

### 1.4. In Combination with Synchrotron X-ray Scattering

Colloidal systems can also be investigated using X-ray scattering, in particular, small-angle X-ray scattering (SAXS) and wide-angle X-ray diffraction (WAXD) [22,23,24]. Unlike DLS, which computes temporal correlation functions of the scattered intensity and is linked to particle size information indirectly via particle diffusion behavior, SAXS and WAXD put emphasis on the spatial correlations in the measured system, and thereby provide direct structural information of the particles in a similar fashion as static light scattering (SLS) does.

The primary goal of our instrument is to combine laser light scattering (LLS) that can perform SLS, DLS, and 3D-PCCF with synchrotron SAXS/WAXD so that *time-resolved simultaneous light and X-ray scattering* experiments can be performed. We have found it advantageous to build the LLS and 3D-PCCF instrument as an independent portable device (without X-rays), so that it can be pre-aligned outside the synchrotron hutch, and thereby also not unnecessarily wasting precious synchrotron beam time with the relatively more difficult 3D-PCCF alignment process. The combination of simultaneous light and X-ray scattering allows us to cover a larger range in length and time scales. In the length scale range, time-averaged scattered intensity measurements can reach from about 1 nm for SAXS and even smaller for WAXD as well as up to microns for SLS or ultra-small angle SAXS (USAXS), while for DLS, the time scale depends partly on how photo-electron pulses are being treated and can easily go down to tens of nanoseconds. At present, we are dealing with standard photoelectron pulses with correlation times down to slightly less than a microsecond. The combination of these features enables the instrument to trace kinetic processes in many fluid systems, including those that *do not permit solution dilution* in order to meaningfully reflect the real-life kinetic changes in systems of interest. For example, the structure and dynamics of amyloid aggregation [25,26], as related to Alzheimer’s disease, can be one of many meaningful examples that fit into this category.

## 2. Results and Discussion

For those less familiar with PCCF, it is recommended that the readers first read 3D-PCCF in Section 3.1.2. in order to be more familiar with the combination of PCCF and SAXS/WAXD.

### 2.1. Considerations for Combined PCCF with SAXS/WAXD—Sample Cell Design

In addition to the instrumental improvements as to be described in the Experimental Section for separate measurements of PCCF and SAXS/WAXD, two further requirements must be satisfied in order to combine the two techniques together. The PCCF set-up itself was designed to be portable and relatively easy to align, and, most importantly, a special sample cell, which should be suitable for both types of measurements, was designed, constructed and tested. It should be noted that the experimental requirements for SAXS/WAXD and PCCF could be quite different (sometimes even opposite), so the design of the sample cell became a challenge and would require careful thought, as well as, invariably, include compromises.

In solution SAXS/WAXD measurements, one has to consider the sample scattering power as well as the X-ray absorption in order to achieve an optimum signal-to-noise ratio. For a plane-parallel plate (which can serve as an approximation for the intersection of a cylindrical cell with a beam of much smaller diameter at small scattering angles), the intensity has the following relationship with sample thickness [23].

(1) I∝d⋅exp(−μd)

where μ is absorption coefficient and *d* is sample thickness. Based on this simple relationship, the intensity change as a function of *d* can be obtained.

**Figure 1:**
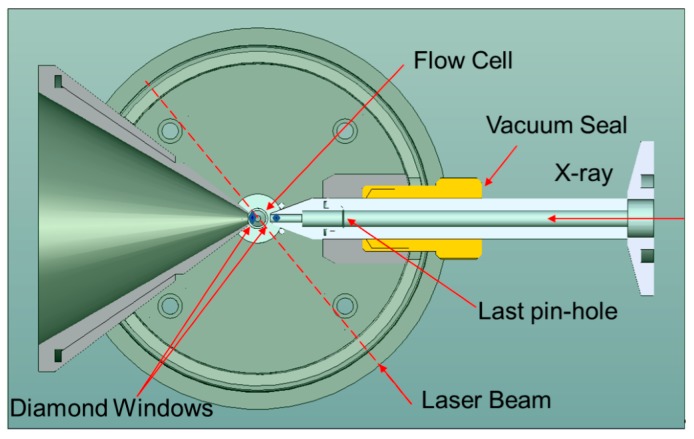
Top view of 2-mm cylindrical flow cell with diamond windows (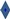) for X-ray scattering measurements and refractive index-matching fluid for light scattering.

In SAXS/WAXD experiments, thin-wall sample capillaries typically have diameters of around 1.0 mm. However, in DLS experiments, a strong reflection due to the small sample cell diameter will be very problematic. A compromise in our combined study is to choose capillary tubing with a 2.0 mm (or up to 5.0 mm) diameter, as shown schematically in Figure 1. Furthermore, DLS measurements require the use of an index-matching fluid. But in SAXS/WAXS measurements, any possible scattering from media other than the testing sample itself should be avoided. To achieve acceptable results for both techniques, the following considerations were integrated into the cell design.

(1) An ultra-thin wall quartz capillary (with 2.0 mm outer diameter) was used. The wall thickness was about 10 μm.
(2) A tube directly connected to the X-ray collimation system ends very closely to the quartz capillary (about 0.3 mm). This tube, together with the collimation system as a whole, could be evacuated. The third pinhole, as described in the Experimental Section, was mounted inside this tube. A diamond window (in blue color, as shown in Figure 1) with an effective aperture diameter of 2.5 mm and a thickness of 0.25 mm was used at the end of the tube. Background scattering before the sample was, therefore, reduced to a large extent.
(3) After the capillary, a receiver cone with the same diamond window (in blue color, as shown in Figure 1) as used in the collimation tube containing the third pinhole was placed very close (about 0.5 mm) to the opposite side of the capillary. The scattered beam, together with the incident X-ray beam, passed through the diamond window into a vacuum chamber before reaching the wire detector.
(4) Water (HPLC grade) was used as an index-matching fluid, which caused less scattering/absorption when compared with silicon oil in the two narrow gaps between the capillary cell and the two diamond windows.

**Figure 2:**
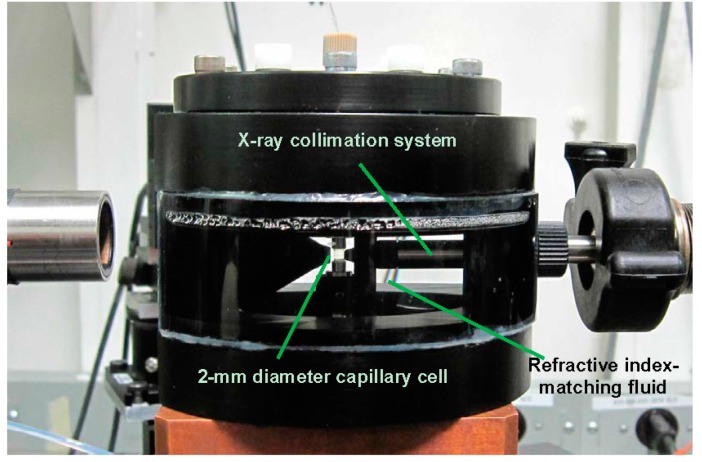
Side view of sample cell with index-matching fluid for combined laser light scattering (LLS) with photon cross correlation function (PCCF) and small angle X-ray scattering/wide angle X-ray diffraction (SAXS)/WAXD study. A 2-mm diameter flow cell was seated in the center. Tube on the right side was connected with the X-ray collimation system. A cone on the left side was used to minimize optical path of scattered X-rays in the medium of refractive index-matching fluid. After the receiver cone for X-rays, the scattered beam passed through a vacuum chamber to reach the wire detector (Not shown here).

**Figure 3:**
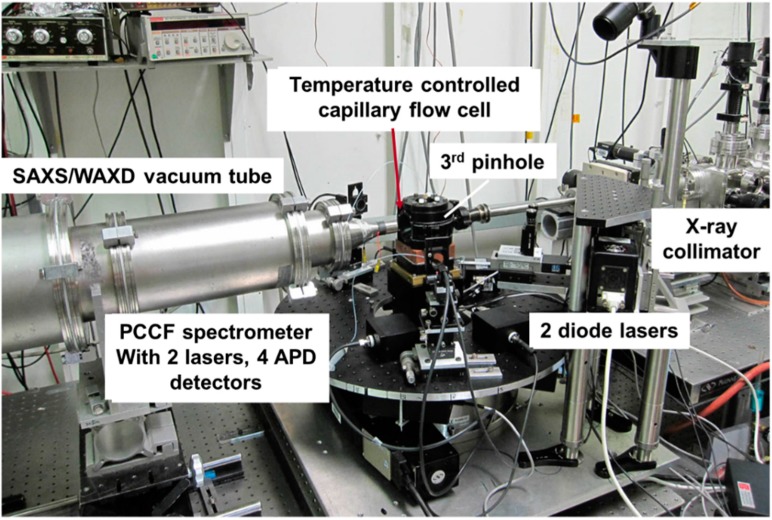
Combined 3D-PCCF spectroscopy and synchrotron SAXS/WAXD set-up.

Figure 2 shows the side view of the scattering chamber, as also presented schematically in Figure 1. Together with the use of microfluidic device (Not shown) for the introduction of solvent, solution and the processes, including dilution and flushing of cell for cleanups, the completed instrumentation for a combination of SLS, DLD, 3D-PCCF and synchrotron SAXS/WAXD is shown in Figure 3.

### 2.2. 3D-PCCS Demonstration

The proper demonstration was carried out in the integrated mode with the 3D-PCCF instrument already aligned with the synchrotron SAXS/WAXD instrument at the X27C, beamline of the National Synchrotron Light Source at Brookhaven National Laboratory (NSLS/BNL). The details of SAXS/WAXD instrumentation will be discussed in the Experimental Section. The demonstration is being presented here in order to illustrate that 3D-PCCF can be incorporated into SAXS/WAXD.

**Figure 4:**
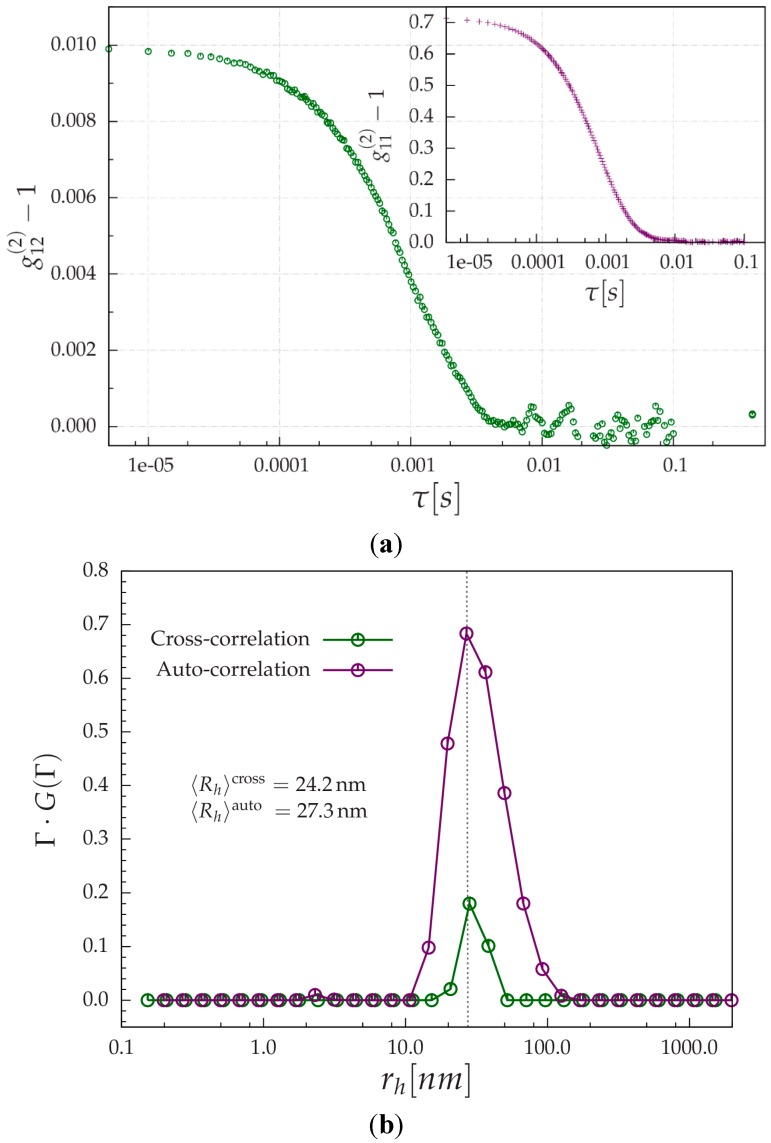
(**a**) Example cross-correlation and autocorrelation function (in-set) of aqueous (*c* = 5 × 10^−4^ g/mL) polystyrene latex standard (nominal diameter = 50 nm). Experiments were performed at θ = 40^o^, using a 2-mm diameter cylindrical flow cell; and (**b**) Apparent hydrodynamic radius (*R*_h_) distribution determined by Laplace inversion [1,2] implemented by the CONTIN program, from auto-correlation and PCCF data. *R*_h_ auto has uncertainty of about 2% while that of cross has uncertainty of 8%. Results were obtained from Figure 4a by using Equations (2) and (3).

Aqueous polystyrene latex standard (nominal diameter = 50 nm, from Polysciences Inc., Warrington, PA, USA) with a concentration of *c* = 5 × 10^−4^ g/mL was tested in both auto-correlation and cross-correlation modes, as shown in Figure 4a. The experiment was performed at a scattering angle θ = 40° at room temperature. The auto-correlation function was accumulated for 2 min, the cross-correlation function for 10 min. At this moment, the beating efficiency (the β-value, as manifested by the intercept on the *y*-axis) in Figure 4a is quite low. It is noted that the theoretical maximum can be 0.25 [18]. The auto-correlation function as shown in the in-set of Figure 4a, had a β-value of about 0.7. It should be pointed out that, with the fiber-optics design, our apparatus should easily be able to achieve β > 0.8 for the auto-correlation function, and can go beyond 0.9 with a little more effort on alignment. However, to succeed in PCCF measurements, we sometime have to sacrifice the efficiency in the auto-correlation function of a single detector and to seek for a closer match of the β-value in using both detectors. Though much higher β-values have been reported, the status of our current apparatus is due to a compromise among various practical considerations for the time being.

There are several ways to improve the beating efficiency. Firstly, one can produce a smaller beam waist, which will effectively increase the coherence area [1,27]. However, in the PCCF method, this invariably leads to extra alignment difficulties, because the β-value tends to decrease in an exponential way due to the spatial mismatch in the scattering volume [13,17]. Secondly, the coherence area can be increased by using a flat cell, where the scattering volume is physically set by the cell thickness [18]. Unfortunately, this method is cumbersome for multi-angle measurements; furthermore, such a flat cell is difficult to construct for simultaneous measurements with X-ray scattering experiments. Thirdly, a fundamental solution to the low efficiency problem in PCCF is to try to differentiate two laser beams. Glatter implemented this method by using two incident beams with orthogonal polarizations. It significantly increased the β-value up to 0.75 [18]. But extra calibration would be required for multi-angle measurements due to the horizontally polarized incident beam (with the scattering plane being horizontal); and the situation will surely become more complicated for solutions containing anisotropic particles. Ian D. Block and Frank Scheffold [28] used acousto-optic modulators to alternatively shutter laser beams at each acquisition instant, implying that the scattered field was produced by only one laser beam. The interference of the second beam was therefore removed. Besides extra optical components, this method would require choice of the right shuttering frequency, so that scattering signals from adjacent data acquisition intervals could be correlated. The first and the third approaches can be implemented with our instrumentation if so desired in the future.

For monodisperse systems, the first-order electric field temporal correlation function (both auto-correlation and cross-correlation function) can be modeled by using a single exponential decay function.

(2) $$g^{\left(1\right)}\left(\tau \right)=\exp\left(-Dq^{2}\tau \right)$$

Symbol *q* (=4π*n*λ^−1^sin(θ/2)) is the absolute value of the scattering vector, τ is the decay time and *D* is the diffusion coefficient, which is related to the hydrodynamic radius of the solute particles via the Stokes-Einstein equation.

(3) $$D=\frac{k_{B}T}{6\pi \eta r}$$

Whilst for the polydisperse system, Equation (2) becomes a summation of multiple exponential decays with a characteristic line width distribution *G*(Γ), where Γ is the characteristic line width [1,29]. By performing Laplace inversion (to derive size distribution from overall correlation function), the hydrodynamic radius (*R*_h_) distribution can be derived, which is shown in Figure 4b. The distribution in Figure 4b was produced by using the constrained regularization method, implemented by the CONTIN program [30,31]. A similar *R*_h_ distribution, peaked at about 27.0 nm was obtained even though the beating efficiency was weak for PCCF. The average values were 24.2 and 27.3 nm, as derived from PCCF and auto-correlation function, respectively, showing a fairly reasonable initial agreement at this stage of development.

### 2.3. Experimental Test on X-ray Instrumentation in the Combined Facility

**Figure 5:**
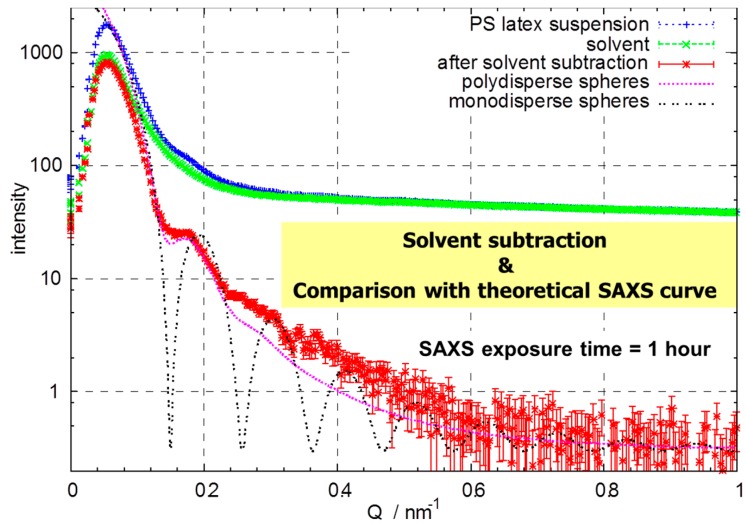
SAXS curve (exposure time equals 1 h) of aqueous suspension (*c* = 1.0 × 10^−4^ g/mL) of polystyrene latex spheres with diameter = 50 nm. Solvent subtraction and comparison with theoretical SAXS curves are also shown. Intensity in relative units.

To test the collimation system and the sample cell, we chose the same 50 nm diameter polystyrene latex aqueous suspension discussed above for the PCCF tests at various concentrations. The data was taken at X27C/NSLS/BNL with a 200 mm × 200 mm multiple wire proportional counter (2D-MWPC) detector made by EMBL. This type of detector has very low background noise characteristics. The data collection time was set to 1 h each for the empty cell, the cell with buffer solution (water) and the latex suspension, respectively. Figure 5 shows the scattering from the suspension and buffer, after solvent subtraction and theoretical SAXS curves. The concentration in the experiment, as shown in Figure 5, was 1 × 10^−4^ g/mL. We can see that at this low concentration, the signal to noise ratio was still acceptable.

## 3. Experimental Section

### 3.1. PCCF Techniques

#### 3.1.1. Cross Correlation and Two-Color Approaches

The cross-correlation function idea was first demonstrated in G. Phillies’ pioneering work where the geometry of a sample being illuminated by two counter-propagating laser beams (with the same color) was proposed. Two detectors were set face-to-face at 90° with respect to the two beams. Photons detected by the two detectors are equivalent in momentum transfer (despite the opposite directions of the beams; momentum transfers depend on the vector difference between incident and scattered beams), and therefore, are correlated when only singly scattered [32,33]. However, his experiment cannot be performed at angles other than 90°. K. Schätzel and then others succeeded in another cross-correlation set-up with high beating efficiency and the capability of performing cross-correlation function measurements at multiple scattering angles [14,34,35,36]. His two-color dynamic light scattering experiment used two incident laser beams with different wavelengths (*i.e.*, colors) on the sample. For this scattering geometry, all incident and scattered beams were laid within the same plane. Since the two beams had different wavelengths, the two detectors should be set at different scattering angles to receive scattered photons with the same momentum transfer. While the two-color method was proven to be capable of obtaining significant beating efficiencies (intercept, or β > 0.6), it had very strict requirements on optical alignment [16]. Subsequently, the 3D cross-correlation method was proposed to preserve the merits of the cross-correlation idea, yet making alignment more feasible in practice [13,14,15,16,17,18,19,20]. Therefore, 3D-PCCF was adopted, as will be described in more detail below. The low-β-value problem in 3D-PCCF design can be potentially improved by modulating laser beams [28], or by introducing orthogonal polarization to them [18], depending on the system being investigated.

#### 3.1.2. 3D Photon Cross-Correlation Function (3D-PCCF)

##### Basic Scheme

Of the various possible photon cross-correlation spectroscopy (PCCS) setups and geometries (two-color, 3D, *etc.*), we decided on a monochrome (one-color) 3D scattering geometry for our PCCS instrument. It is noted that from here on we have used the symbols PCCS and PCCF interchangeably. A more detailed description is provided in order to give the reader an easier access to the essence of the theory behind the instrumentation. In this respect, the LS Instruments, Inc. website is highly recommended; Fribourg has provided excellent reference materials on many aspects of light scattering [37].

**Figure 6:**
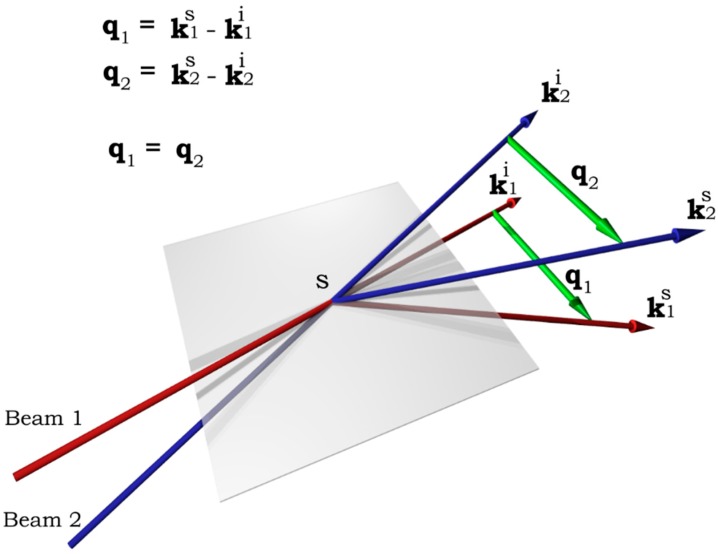
Illustration of the 3D two-beam photon cross-correlation geometry.

The scattering geometry is illustrated in Figure 6. Two beams are symmetric with respect to the central plane and are crossed at a point S. *k^i^*_1_ and *k^i^*_2_ are the wave vectors of the incident beams 1 and 2, respectively; *k^s^*_1_ and *k^s^*_2_ are those of the scattered light, respectively. *q*_1_ and *q*_2_ are the momentum transfer vectors (scattering vectors) of the first and the second beam-detector pair, respectively. *q*_1_ = *q*_2_ is the prerequisite so that cross-correlation can be established.

The normalized second-order intensity temporal cross-correlation function g^(2)^_12_(τ) is related to the first-order electric field temporal cross-correlation function g^(1)^_12_(τ) by

(4) $$g_{12}^{\left(2\right)}=\frac{<I_{1}\left(0\right)I_{2}\left(\tau \right)>}{<I_{1}><I_{2}>}=1+\beta {\left|g_{12}^{\left(1\right)}\left(\tau \right)\right|}^{2}$$

With

(5) $$g_{12}^{\left(1\right)}\left(\tau \right)=\frac{<E_{1}\left(0\right)E_{2}^{*}\left(\tau \right)>}{<E_{1}><E_{2}^{*}>}$$

where *E* is the electric field. β is the *overall* coherence factor, which includes factors such as the beating efficiency due to coherence, the overlap of the scattering volumes, as well as intensity ratios of single scattering and alignment [13,14,15,16,17,18]. When compared with single scattering experiments, the theoretical limit of β for this 3D-PCCS setup is *one-fourth* of that for single scattering [18].

g^(2)^_12_(τ) is the quantity measured in the PCCS experiment by a digital correlator, based on the signals captured by the two detectors responsible for the scattered intensities with momentum transfers *q*_1_ and *q*_2_, respectively. Equation (4) is the Siegert relation in its cross-correlation version, which relates the second order and first order temporal cross-correlation functions.

##### Current Apparatus Specification

**Figure 7:**
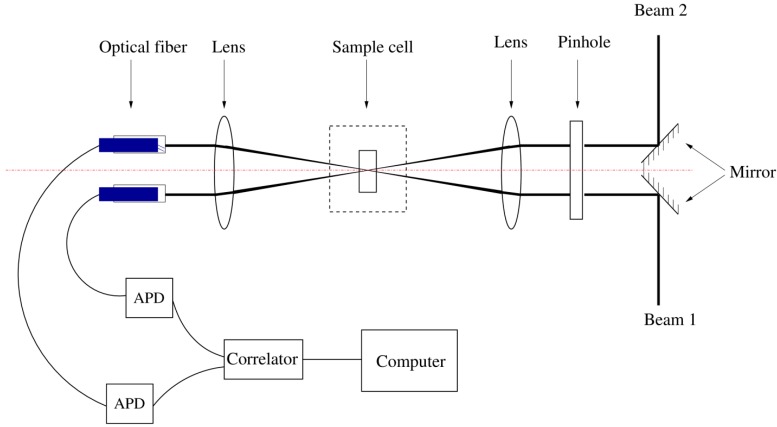
Schematic 2D layout of the PCCS apparatus. The two lasers are set face-to-face with 10 mm off-set. APD stands for avalanche photodiode. To facilate visulization, optical layout is shown in side view, whilest two laser beams are shown in top view.

A 2D layout of the 3D-PCCF apparatus is schematically shown in Figure 7. Laser beams were generated by two individual 30 mW CW diode lasers (Mini-L30, Brookhaven Instruments Inc., Holtsville, NY, USA), with the wavelength being 637 nm. The transverse mode was TEM_001_. The lasers were precisely stabilized at 16.5 ± 0.01 °C, with power fluctuations being within 0.5%. The polarization ratio at 637 nm was larger than 150:1. The two lasers were compact in size: 94 mm × 70 mm × 112 mm. The initial beams were elliptical, 2 mm × 3 mm in size. The two beams were separated by 10 mm. Two mirrors were placed such that the reflected beams could proceed parallel to each other and in the same vertical plane.

Two 1.0 mm diameter pinholes were used to re-shape the elliptical beams to the round shape. Subsequently, the beams passed through a lens with 175 mm focal length, refracting the two beams to the central point of the sample cell and focusing them down to 140 μm in diameter at that central point, according to the following formula for Gaussian beams (aberration was neglected).

(6) $$w_{0}=f\frac{\lambda }{\pi w_{1}}$$

where *w*_0_ is the radius of the beam waist, *f* is the focal length of the lens, λ is the wavelength and *w*_1_ is the radius of the incident beam.

The cylindrical sample cell could be varied between 2 to 5 mm in diameter, made up of quartz with a wall thickness of 10 μm, and seated in a glass cylinder with 60 mm diameter, filled with refractive index-matching fluid. A beam stop was set in the glass cylinder to trap incident beams after the sample. The scattered light would pass through the second lens with the same focal length and the same distance to the center of the sample cell as the first lens, and then were guided via two optical fibers separated by 10 mm to two Avalanche Photo Diodes (APDs). The cross-correlation function was computed by a digital correlator (TurboCorr, Brookhaven Instruments Inc.) and was sent to a computer for further processing.

The optical fibers and the second lens were mounted in a precision motorized rotation stage (RV350PP, Newport Corporation, Stratford, CT, USA). The detection range could go from 3° to 160°. When combined with the synchrotron X-ray scattering experiment, and in order to achieve the designed angular detection range, two pairs of optical fibers and detectors were used to solve the geometrical restrictions caused by the X-ray beam paths, especially the cone for the scattered X-ray beams, which all had to be in vacuum.

##### Geometrical Considerations for Combined 3D-PCCF and Synchrotron SAXS/WAXD

**Figure 8:**
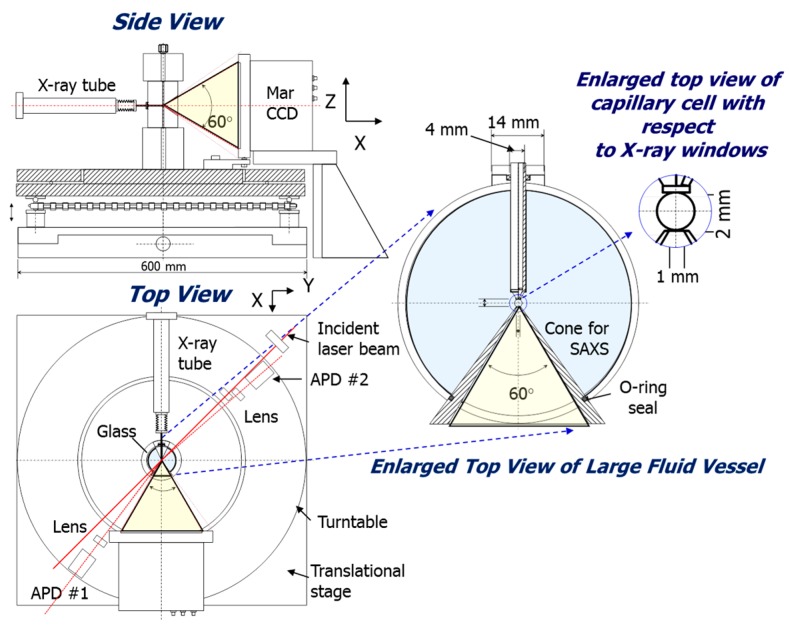
Side and top views of X-ray pathways.

**Figure 9:**
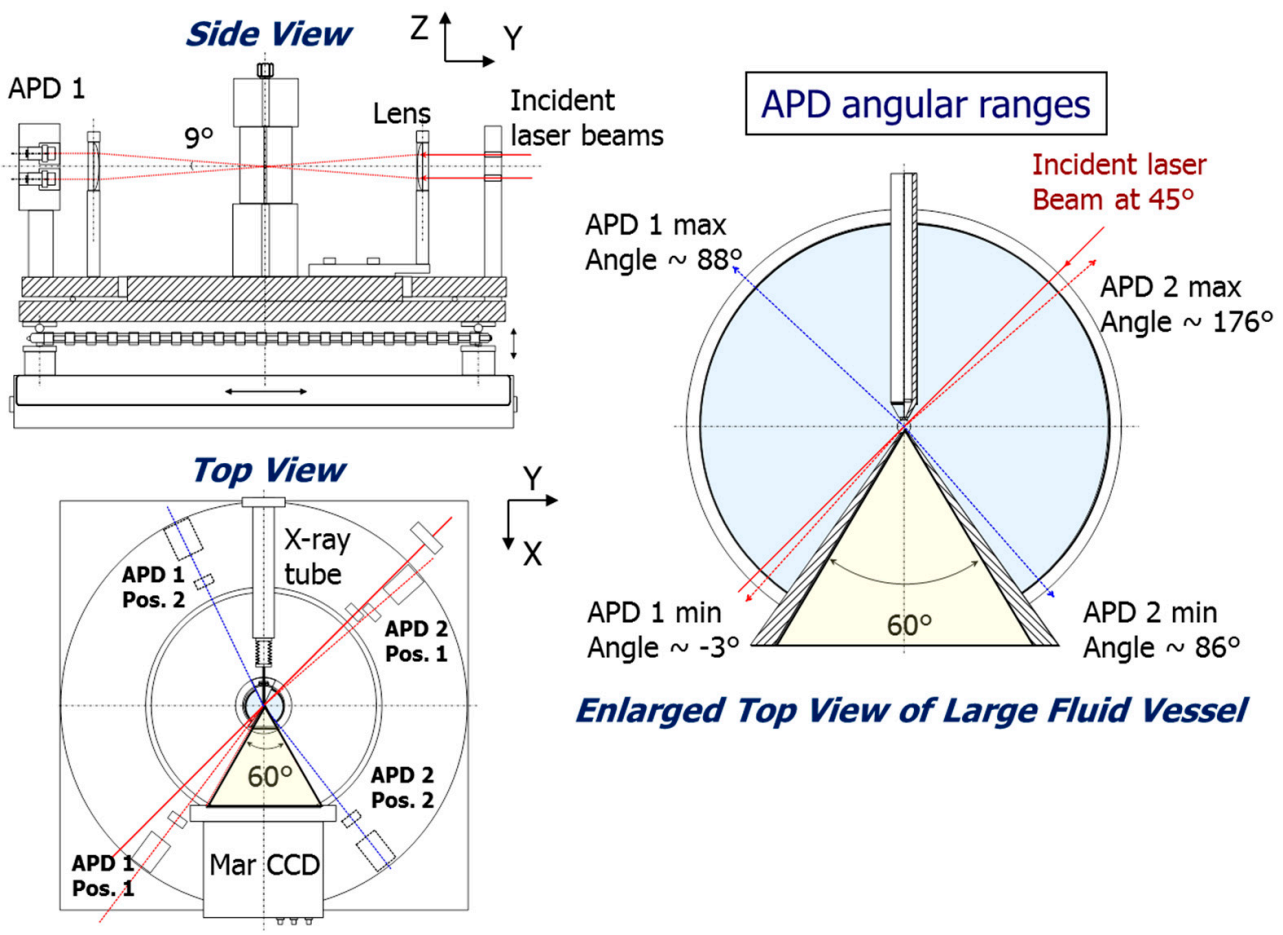
Side, top, and enlarged top views of laser beam pathways. It should be noted that from the side view, APD 1 has two APD detectors and APD 2 has also two APD detectors (Not shown). From the top view, APD 1 can move from position (Pos.) 1 to position (Pos.) 2, illustrating the ranges that can be covered by APD 1. While APD 2 can move from the corresponding positions 1 and 2, covering the APD angular ranges as shown in the enlarged view.

The key points in providing Figure 8 and Figure 9, showing the X-ray and laser beam pathways, are to demonstrate that it is feasible to combine 3D-PCCF with synchrotron X-ray scattering; and in essence, without sacrificing the scattering ranges for 3D-PCCF, DLS and SLS, as well as SAXS/WAXD, covering the length scales down to the crystal spacing in X-ray diffraction and the laser light scattering range reaching to a few degrees in θ, as well as for θ close to 180°, thereby extending the accessible length scale range from Angstroms to microns. Overlapping of light scattering *q*-range with the SAXS *q*-range could be achieved by taking advantage of synchrotron X-rays that permit variations in the incident X-ray wavelength.

With the use of photon detectors in two separate quadrants for laser light scattering, as shown in Figure 9, dissymmetry ((θ < π/2)/(θ > π/2)) measurements of scattered intensity, say at scattering angles of 45° and 135°, could be measured over short-time intervals, corresponding to an *additional capability* of monitoring kinetic studies on the changes in particle size as a function of time down to the millisecond range, provided that the count rate could be sufficiently high.

##### Diagrams and Photographs of 3D-PCCF Components

**Figure 10:**
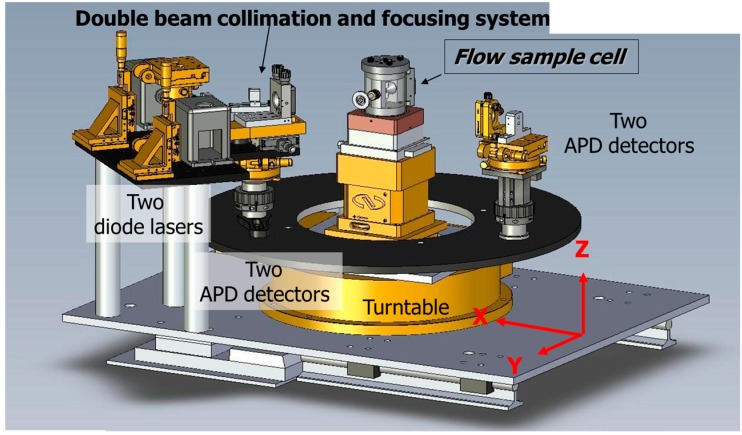
Schematics of essential components for 3D-PCCF. Only two APD detectors are shown schematically in the figure, with the other two being partially blocked by the beam collimation and focusing system.

**Figure 11:**
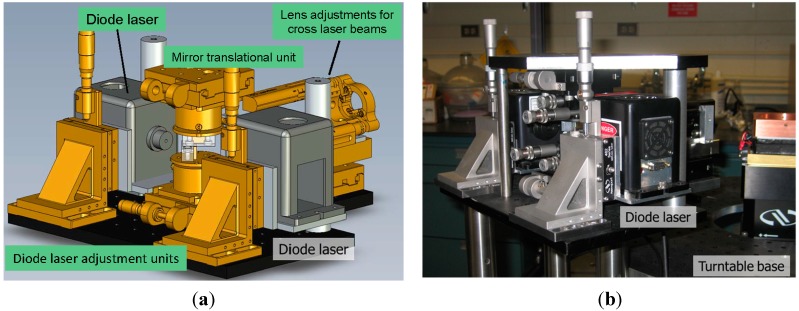
(**a**) Dual laser and focusing unit; (**b**) Photograph of dual laser and focusing unit.

In order to establish an independent 3D-PCCF instrument but with the capability for synchrotron SAXS/WAXD measurements, the entire light scattering unit should have its own platform. In addition, the 3D-PCCF spectrometer should then be able to move into the synchrotron X-ray set-up with precision alignment by remote control since the synchrotron X-ray beam is defined by its own geometry. Figure 10 shows the schematics of essential components for 3D-PCCF, including two diode lasers in combination with the focusing system (as shown in Figure 11) and two pairs of APD detectors. The entire platform of the 3D-PCCF instrument was mounted on a platform with precision translational controls in the x and z directions, where x was the direction of the synchrotron X-ray beam and z was the vertical direction. The thermally isolated sample cell mount was fixed on the 3D-PCCF instrument platform with the flow cell adjusted to be at the center of rotation of the remote controlled turntable.

A flow cell was used because of the precision requirement to center the sample cell in coincidence with the center of rotation of the turntable. For a sample cell with 2-mm diameter, it is not advisable to change the sample cell during each set of measurements after alignment. Thus, the flow cell was connected with a microfluidic device (Not shown) that could be used to wash the sample cell; to introduce the solvent for background calibration; to dilute polymer solutions; and to introduce the polymer solution of interest into the flow sample cell. With synchrotron X-rays, one may wish to consider possible radiation damage due to intense X-rays. The flow cell can then be used by circulating sample solutions at different flow rates [38].

Figure 11a,b show more details of the dual laser beam and focusing unit, which has its own platform and is decoupled from the turntable where the two sets of APD detectors sit. The incident dual laser beam and focusing unit was designed to be compact, not only for its own stability but also to reduce the size of the 3D-PCCF instrument to a compact format for easier adaptation to different synchrotron X-ray setups.

##### 3D-PCCF Alignment

Successful observation of cross-correlated single scattering while suppressing multiple scattering is strongly dependent on the optical alignment. The key point, as has been briefly explained above, is to have the two detectors to capture light that is scattered from exactly the same scattering volume element with exactly the same momentum transfer (in both magnitude and direction). This requirement has two consequences in practice:

(1) The two laser beams must be precisely crossed at the center of the sample cell. Any mis-alignment of the beams will not only cause the two momentum transfer vectors not to be identical but also to reduce the overlapping region of the two beams, causing various inefficiencies for the cross-correlation function.
(2) The two detectors should look at exactly the same speckle. It is often observed that the two detectors can achieve a good beating efficiency in the auto-correlation mode while generating only a poor cross-correlation signal. Assuming that the beams are well crossed, this is likely caused by the two detectors looking at different speckles with independent temporal correlations.

For the beam alignment, a needle (made by tungsten-cobalt alloy, 70 microns in diameter) held in a precision tubing (stainless steel, 0.5 mm outer diameter), was placed at the center of the sample cell, *i.e.*, at the rotational axis of the turntable. The two beams, when properly crossed at the same point, will generate two identical diffraction patterns caused by the cylindrical object that is the needle. Fiber optic alignment turned out to be very critical. Two fiber holders were designed such that each of them had two translational degrees of freedom (vertical and horizontal, while the distance between the fibers and the second lens is less important), and could be tilted in three directions. This guaranteed that orientation of the two fibers could be tuned independently.

### 3.2. Synchrotron SAXS Measurements

#### 3.2.1. Upgrade of Collimation System for SAXS/WAXD

Synchrotron radiation has great strength in X-ray studies because of its high brightness and small divergence angle. For SAXS studies, a very well defined X-ray beam and the absence of parasitic scattering, *i.e.*, the quality of the collimation system, are crucial. There are various approaches to collimate a SAXS system. A flexible approach employed by many synchrotron beamlines is to utilize a series of (horizontally and vertically) adjustable slits to collimate the beam. However, the complete control of all the slits in such a slit-based collimation system involves a large number of translational, tilt and rotational movements. The difficulty in aligning such a system is not only the large number of adjustable parameters but also that the adjustments of individual slits are often not independent from each other but coupled. Thus, previously aligned slits often need to be revisited during the alignment process, making it a tedious and lengthy process. Unlike typical laboratory SAXS systems where the geometry of the X-ray source is fixed by the manufacturer and, therefore, the collimation system can be fixed with minimal need for re-alignment, the optics of a synchrotron beam will always have small variations due to electron orbit changes, temperature variation of mirrors upstream in the synchrotron beam pathway, and many other effects. These small changes are typically large enough to cause a frequent need to re-align the collimation system. Thus, a simple collimation system with user-friendly alignment control software is desirable.

**Figure 12:**
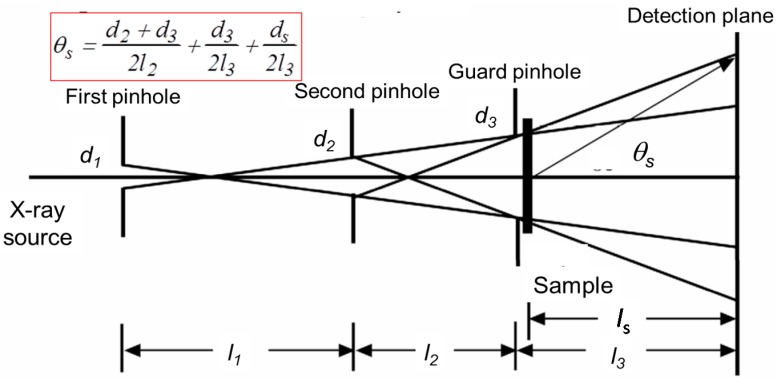
Three-pinhole collimation system for X-ray scattering. *d*_i_ denotes diameter of pinhole i; *I*_i_ denotes the distance between pinholes, with *I*_3_ denoting the distance between the guard pinhole and the detection plane, while *I*_s_ denotes the distance between the sample and the detection plane. θ_*s*_ is the scattering angle between the beam center of the sample and the point of detection.

The collimation system that we constructed is based on a 3-pinhole configuration, which has previously been proven to work very well at beam line X27C, NSLS/BNL, improved for robustness and ease of alignment. The new collimation system consists of three pinholes (Figure 12), where the first and the second pinholes define the cross-section shape of the synchrotron beam, while the third pinhole (PH3) acts as a guard pinhole, blocking any parasitic scattering generated by the edge of the second pinhole [39,40]. The pinhole system was designed as an overall rigid object (Figure 12, for the current application, the third pinhole in the new unit was attached to the 3D-PCCF instrument). The vacuum tubings (38.1 mm OD) were hard connected through flanges and were reinforced by two aluminum 96 mm × 96 mm optical rails, sitting on an optical table. The first pinhole was configured such that it was seated on the pivot point of the whole rigid structure. The second pinhole was likewise seated on a pivot point with *x*- and *z*-motion freedom.

**Figure 13:**
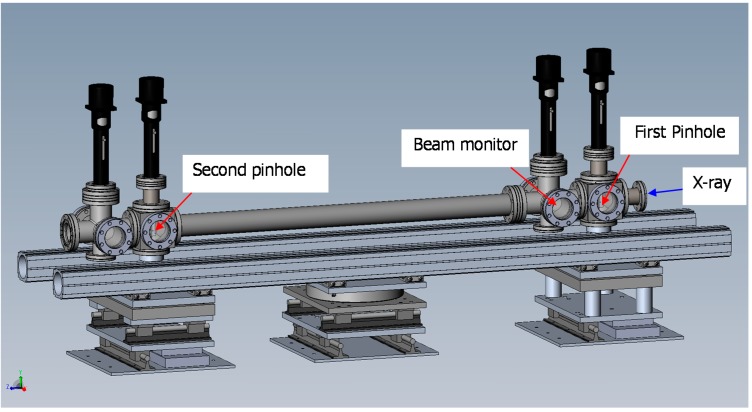
Schematics of three pinhole system with the third pinhole to be incorporated into the 3D-PCCF instrument. Distance between first and second pinholes = 1105 mm.

**Figure 14:**
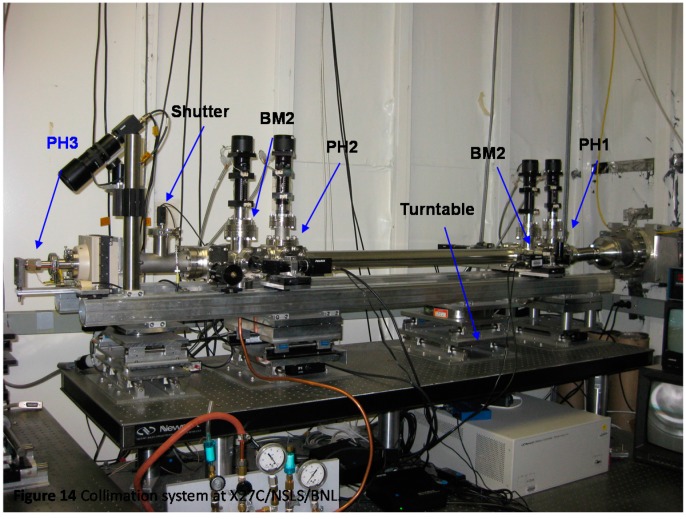
Collimation system at X27C/NSLS/BNL.

In Figure 13, the two pinholes could be moved independently in the vertical (*y*-motion) direction. Each pinhole assembly, except for the last guard pinhole, consisted of a tantalum plate that has three pinholes with different diameters, mounted on a pair of cross-roller bearing slides, so that it could be lifted or lowered to choose a designated combination of pinhole diameters by a precision actuator. In between the gate valve and the last pinhole, there was a rotary solenoid shutter with a pin-diode mounted in the center of the shutter. This shutter could be remotely controlled to swing in/out of the synchrotron beam path. The beam intensity after the first and second pinholes could be measured using this pin-diode when the rotary shutter was in the beam path. The whole system was mounted on a 24 in × 72 in × 4 in optical table and the system was pre-aligned before moving it to the X27C/NSLS/BNL station. The collimation system could be easily moved and reinstalled at future locations, e.g., at NSLS-II/BNL. Figure 14 shows a photograph of the complete collimation system in the synchrotron hutch.

#### 3.2.2. Collimation Control Procedures

The translational stages and the actuators (eight in total, six for the three pinhole *x*- and *y*-movements, and two for beam monitor *y*-movement) were driven by a control unit (Newport XPS-8), and were connected to a controlling computer through a standard TCP/IP port. The graphical web front-end allowed the user to view up to eight connected video cameras, including but not limited to the beam monitors after the first two pinholes, and featured optional cross-hairs to mark positions of interest on the video images. The beam intensity could be automatically measured and visualized while horizontally or vertically scanning over a pinhole in order to maximize the synchrotron beam intensity passing through it.

The collimation alignment procedure was simple, since only six stages of movement were involved. The step size for the scanning was set to 10 μm. The quality of the alignment can be checked by small angle scattering from air. The SAXS system at X27C/NSLS/BNL consists of a vacuum flying tube up to 2000 mm in length, a choice of lead beam stops with 2.5 mm being a typical beam stop diameter, and various detection systems, including MarCCD, Fuji imaging plate and 1D and 2D wire detectors. Upon successful alignment, the primary beam could be evenly covered by the beam stop, and there were no noticeable streaks from parasitic scattering. The optimum pinhole positions were not coupled but independent, starting with the first pinhole, so that, thanks to the automated pinhole scanning, the total alignment procedure could be accomplished very quickly, typically less than an hour and sometimes only minutes.

### 3.3. Cell for Liquid Samples

**Figure 15:**
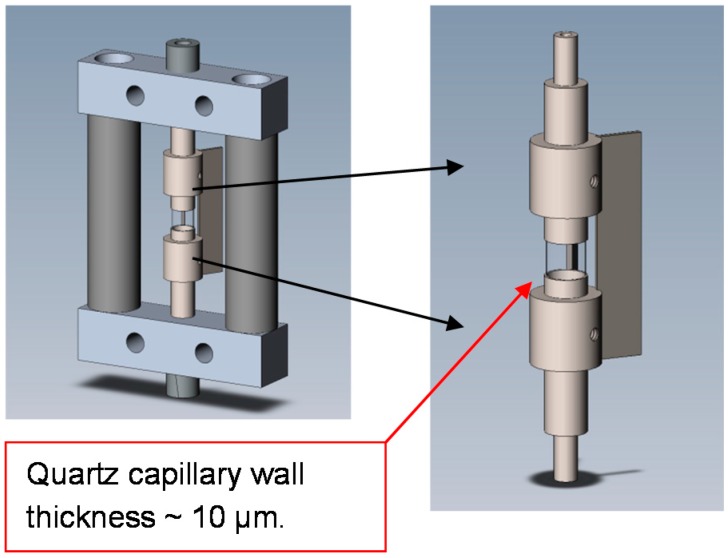
Flow cell structure.

Biological samples in aqueous solution pose a challenge to X-ray studies, since the electron density differences between the buffer solution and the solute of interest are typically very small, leading to poor signal-to-noise ratios. The scattered intensity depends on the square of the electron density difference, and the signal-to-noise ratio in terms of its standard deviation depends on the square root of the total scattering intensity accumulated over time. In order not to further deteriorate the signal-to-noise ratio by additive backgrounds or absorption, one has to minimize the air scattering before the synchrotron beam entering the sample, and reduce the X-ray absorption by the wall of the sample cell as much as possible [23,38]. We chose a 2 mm diameter quartz tube with ultrathin (10 μm) walls and cut it into 5 mm long segments to be used in a stop/flow cell. The cell volume was 16 μL, excluding the volume of the inlet and exit plastic tubing connected to the cell (of which the inner diameter of the plastic tubing for fluid transport could be as small as 0.15 mm in diameter). Figure 15 shows the structure of the cell. The quartz capillary was sealed in stainless steel adapters made from precision stainless steel tubing. The two adapters were fixed in a pair of sleeves, which were connected and reinforced by two fins made of a stainless steel plate. This configuration makes it relatively easy to replace of the quartz tubing, should it become necessary. To reduce the air scattering before the sample, a cone-shaped stainless steel tubing was attached to the last pinhole. The cone shaped tube had a diamond window (0.25 mm thick, 3 mm diameter) on its exit tip, as shown in Figure 1 and Figure 2, and the detail view in Figure 8. During the experimental setup procedure, the sample cell was carefully moved close to the diamond window. The angle of the cone and the fins was matched such that when they were in contact, there would be a gap of about 0.1 mm between the surface of the diamond window and the outer wall of the quartz capillary. The cell shown in Figure 15 was for X-ray studies only. To combine with light scattering, the central quartz capillary (together with adapters and fins) needs to be mounted in the cylinder filled with refractive index matching fluid (Figure 2).

## 4. Conclusions

The goal of this project was to combine SLS, DLS and 3D-PCCF with synchrotron SAXS/WAXD in order to investigate multiple length scale structures, including dynamics and kinetics of temporally evolving solutions of colloids, polymers, or bio-macromolecules. We expect the new instrument to be promising for many applications, such as deciphering protein aggregation processes.

A portable 3D-PCCF apparatus has been built which is able to measure particle dynamics and collect particle size information in both dilute and mildly turbid solutions. The synchrotron X-ray collimation system of beam line X27C at the NSLS at BNL was upgraded and outfitted with a user-friendly remote control interface. The new collimation system is able to generate a high quality beam while minimizing unwanted background or parasitic scattering. The design of the collimation system in combination with the remotely accessible collimation control program allows for a rapid and semi-automated alignment procedure. Standard samples of aqueous polystyrene latex suspensions were tested separately by both SAXS and PCCF. Both measurements yielded satisfactory information. A new sample cell was designed and constructed to be suitable for simultaneous SLS/DLS or 3D-PCCF and SAXS/WAXD studies. Aside from the Glatter approach, the 3D-PCCF can also be improved by temporarily separating the two scattering experiments from the two separate laser beams with modulation of the incident laser beams and gating the detector outputs at frequencies exceeding the timescale of the system dynamics.

The two techniques are now ready to be combined to perform time-resolved experiments and experiments involving local changes in dynamical properties as well as kinetics of processes. The instrument was designed in anticipation of the transfer from NSLS to other synchrotron facilities, permitting very minor modifications in terms of X-ray beam height and extension of higher resolutions by extending the pinhole separation distance.
